# Phenotype and envelope gene diversity of *nef*-deleted HIV-1 isolated from long-term survivors infected from a single source

**DOI:** 10.1186/1743-422X-4-75

**Published:** 2007-07-16

**Authors:** Lachlan Gray, Melissa J Churchill, Jasminka Sterjovski, Kristie Witlox, Jennifer C Learmont, John S Sullivan, Steven L Wesselingh, Dana Gabuzda, Anthony L Cunningham, Dale A McPhee, Paul R Gorry

**Affiliations:** 1 Macfarlane Burnet Institute for Medical Research and Public Health, Victoria, Australia; 2 Department of Microbiology and Immunology, University of Melbourne, Parkville, Victoria, Australia; 3 Department of Medicine, Monash University, Melbourne, Victoria, Australia; 4 Department of Pathology and Immunology, Monash University, Melbourne, Victoria, Australia; 5 Australian Red Cross Blood Service, Sydney, New South Wales, Australia; 6 Faculty of Medicine, University of Sydney, Sydney, New South Wales, Australia; 7 Dana-Farber Cancer Institute, Boston, MA, USA; 8 Department of Neurology, Harvard Medical School, Boston, MA, USA; 9 Westmead Millennium Institute, Westmead, New South Wales, Australia; 10 National Serology Reference Laboratory, St. Vincent's Institute for Medical Research, Fitzroy, Victoria, Australia

## Abstract

**Background:**

The Sydney blood bank cohort (SBBC) of long-term survivors consists of multiple individuals infected with attenuated, *nef*-deleted variants of human immunodeficiency virus type 1 (HIV-1) acquired from a single source. Long-term prospective studies have demonstrated that the SBBC now comprises slow progressors (SP) as well as long-term nonprogressors (LTNP). Convergent evolution of *nef *sequences in SBBC SP and LTNP indicates the *in vivo *pathogenicity of HIV-1 in SBBC members is dictated by factors other than *nef*. To better understand mechanisms underlying the pathogenicity of *nef*-deleted HIV-1, we examined the phenotype and *env *sequence diversity of sequentially isolated viruses (n = 2) from 3 SBBC members.

**Results:**

The viruses characterized here were isolated from two SP spanning a three or six year period during progressive HIV-1 infection (subjects D36 and C98, respectively) and from a LTNP spanning a two year period during asymptomatic, nonprogressive infection (subject C18). Both isolates from D36 were R5X4 phenotype and, compared to control HIV-1 strains, replicated to low levels in peripheral blood mononuclear cells (PBMC). In contrast, both isolates from C98 and C18 were CCR5-restricted. Both viruses isolated from C98 replicated to barely detectable levels in PBMC, whereas both viruses isolated from C18 replicated to low levels, similar to those isolated from D36. Analysis of *env *by V1V2 and V3 heteroduplex tracking assay, V1V2 length polymorphisms, sequencing and phylogenetic analysis showed distinct intra- and inter-patient *env *evolution.

**Conclusion:**

Independent evolution of *env *despite convergent evolution of *nef *may contribute to the *in vivo *pathogenicity of *nef*-deleted HIV-1 in SBBC members, which may not necessarily be associated with changes in replication capacity or viral coreceptor specificity.

## Background

The Sydney blood bank cohort (SBBC) of long-term survivors (LTS) consists of multiple individuals who became infected with an attenuated strain of HIV-1 via contaminated blood products from a common blood donor between 1981 and 1984 [1-3]. Viral attenuation has been attributed to gross deletions in the *nef *and *nef*/long-terminal repeat (LTR) overlapping regions of the HIV-1 genome. Despite being infected from a single source, long-term prospective studies on SBBC members demonstrated that the cohort now consists of subjects with slow disease progression (SP), as well as individuals who remain true long-term nonprogressors (LTNP) and antiretroviral therapy-naive with stable CD4 counts and low or undetectable HIV-1 RNA levels [4]. Three SBBC members (one SP and two LTNP) have since died from causes unrelated to HIV-1 infection [3,4]. Although the cohort members had differing clinical courses, a comprehensive longitudinal analysis of *nef*/LTR sequences in the SBBC donor and four of the transfusion recipients demonstrated a convergent pattern of *nef *sequence evolution, characterized by progressive sequence deletions evolving toward a minimal *nef*/LTR structure that retains only the key sequence elements that are required for viral replication [4]. Thus, HIV-1 pathogenicity in SBBC members is dictated by viral and/or host determinants other than those that impose a unidirectional selection pressure on the *nef*/LTR region of the HIV-1 genome.

The HIV-1 *env *gene, which encodes the viral envelope glycoproteins (Env) is a significant viral determinant in HIV-1 pathogenesis [reviewed in [5-7]]. HIV-1 Env initiates viral entry via binding to CD4 and subsequently to a coreceptor, either CCR5 [8-12] or CXCR4 [13]. CCR5-using (R5) HIV-1 strains predominate at early, asymptomatic stages of infection. In 40–50% of infected adults, progression of HIV-1 infection is accompanied by a switch in coreceptor specificity to HIV-1 variants able to use CXCR4 or both CCR5 and CXCR4 for entry (X4 or R5X4 strains, respectively) [14,15]. A switch in the specificity of HIV-1 Env from R5 to X4 or R5X4 is considered an indicator of poor prognosis, partly because it increases the number of CD4+ cells that are susceptible to cytolytic infection by HIV-1, and is associated with rapid progression of HIV-1 infection. R5 HIV-1 variants are present exclusively in the remaining 50–60% of infected individuals who progress to AIDS, without switching coreceptor specificity [16,17], and exert pathogenic effects that contribute to HIV-1 progression via mechanisms that remain poorly understood [5]. Thus, changes in HIV-1 *env *that affect viral tropism are important for progression of HIV-1 infection.

Analysis of inter- and intra-host evolution of *env *sequence has provided important insights relevant for HIV-1 transmission and progression. While several reports showed an inverse relationship between the rate and extent of viral diversification and progression of HIV-1 infection [18-25], other studies demonstrated that disease progression is associated with increasing rates of viral diversity [26-28]. A later study made significant headway in reconciling these conflicting studies by identifying 3 distinct phases of HIV-1 *env *sequence diversity and divergence during the asymptomatic period preceding the development of AIDS [29]; an early phase of variable duration with linear increases (approximately 1% per year) in both viral divergence and diversity; an intermediate phase characterized by a continued increase in viral divergence but with a stabilisation or decline in viral diversity; and a late phase characterized by a stabilisation of viral divergence and a continued stability or decline in viral diversity. The emergence of X4 HIV-1 variants often coincided with transition between the early and intermediate phases. More recent studies identified convergent sequence evolution in *env *during the early phase toward a common ancestral sequence [30], suggesting that HIV-1 recovers certain ancestral features early in HIV-1 infection that most likely serve to restore viral fitness. However, other studies examining HIV-1 progression in individuals harbouring only R5 variants showed an increase in viral diversity in viral isolates obtained from patients with AIDS compared to isolates from asymptomatic individuals [31], raising the possibility that selection pressures driving HIV-1 evolution may be distinct in patients who maintain R5 viral variants compared to those who experience a coreceptor switch.

While the viral determinants underlying the pathogenicity of *nef*-deleted HIV-1 strains harbored by SBBC members are presently unknown, several lines of evidence support the hypothesis that evolution of HIV-1 *env *contributes to disease progression in this cohort; 1) compartmentalized evolution of HIV-1 V3 *env *sequence in cerebrospinal fluid (CSF) of the SBBC donor was shown to contribute to the development of HIV-associated dementia (HIVD) [32]; 2) enhanced cell killing in *ex vivo *human tissue cultures by HIV-1 isolates from the same SBBC subject was predicted to result from more efficient coreceptor usage [33]; and 3) increased Env-mediated fusion was shown to increase the *in vivo *pathogenicity of *nef*-deleted simian immunodeficiency virus (SIV) [34].

To better understand the role of HIV-1 *env *in the pathogenesis of *nef*-deleted HIV-1 strains harbored by SBBC members, we examined the phenotype and *env *sequence diversity of sequential viruses isolated from 3 SBBC members. Isolates from the SBBC "donor" (subject D36; SP) were R5X4 phenotype and replicated to low levels in peripheral blood mononuclear cells (PBMC). In contrast, isolates from 2 SBBC "recipients" (subjects C98 and C18; SP and LTNP, respectively) were CCR5-restricted with variable replication kinetics. Analysis of *env *by V1V2 and V3 heteroduplex tracking assay, V1V2 length polymorphisms, sequencing and phylogenetic analysis showed distinct intra- and inter-patient *env *evolution. Thus, independent evolution of *env *despite convergent evolution of *nef *may contribute to the *in vivo *pathogenicity of *nef*-deleted HIV-1 in SBBC members, which may not necessarily be associated with changes in replication capacity or viral coreceptor specificity.

## Results and discussion

### Subjects

The clinical history of the study subjects, results of laboratory studies and antiretroviral therapies have been described in detail previously [3,4,32]. The results of laboratory studies relevant for the HIV-1 isolates used in this study are summarized in Table 1. Briefly, the SBBC donor, subject D36 presented with HIVD in December 1998 after being infected with HIV-1 for 18 years without antiretroviral therapy. The development of HIVD coincided with a fall in the CD4 cell count to <200 cells/μl and the presence of high plasma and CSF HIV-1 RNA levels. The subject was placed on a highly active antiretroviral therapy (HAART) regimen of abacavir, nevirapine and zidovidine in January 1999, which suppressed plasma and CSF viral loads to below detectable levels and resolved the symptoms of HIVD [32,35]. As reported previously, transfusion recipient C98 commenced HAART in November 1999 after 16 years of HIV-1 infection, following a steady decline in CD4+ T-cells and a gradual increase in HIV-1 RNA from below detectable levels to 1500 RNA copies/ml [3,4]. He died in March 2002 of amyloidosis, which was not HIV-1 related. Likewise, transfusion recipient C18 died in 1995 of causes unrelated to HIV-1 infection. C18 remained antiretroviral therapy naive despite 12 years of infection, with stable CD4 cell counts and low plasma HIV-1 RNA levels [3]. Thus, subjects D36 and C98 showed evidence of slow progression while subject C18 remained a long-term nonprogressor.

**Table 1:** Subjects and laboratory studies

Subject	**Date of infection**	**Date of blood sample**	**Virus name**	**CD4+ T-cell count (cells/μl)^a^**	**Viral load (RNA copies/ml)^b^**	**Antiretroviral drugs^c^**	**Status of HIV-1 progression^d^**
D36	01/1981	07/1995	D36E	552	1500	ABC, AZT, NVP (1/1999–9/2004)	SP
		01/1999	D36L	160	9900	ABC, NVP, 3TC (9/2004-present)	
C18	08/1983	01/1992	C18E	690	N/A	None	LTNP
		03/1994	C18L	809	2805		
C98	01/1982	07/1993	C98E	880	N/A	d4T, NVP, IND (11/1999-death)	SP
		11/1999	C98L	585	BD		

^a^CD4+ T-cell levels were measured by flow cytometry.

^b^Plasma HIV-1 RNA was measured using COBAS AMPLICOR monitor version 1.0 (Roche Molecular Diagnostic Systems, Branchburg, N.J.) prior to July 1999 and version 1.5 after July 1999. HIV-1 RNA levels of <400 copies/ml (version 1.0) or <50 copies/ml (version 1.5) were considered below detection. BD, below detection; N/A, not available.

^c^ABC, abacavir; AZT, zidovudine; NVP, nevirapine; 3TC, lamivudine; d4T, stavudine; IND, indinavir.

^d^SP, slow progressor; LTNP, long-term nonprogressor. Comprehensive laboratory data collected since 1993 and detailed clinical history of the study subjects has been published previously [3, 4, 32], which was used to classify subjects as SP or LTNP.

HIV-1 isolated from PBMC on 2 consecutive occasions was used in this study (Table 1). The time between isolations ranged from 2 to 6 years. For the purpose of this report, the initial isolates are referred to as "early" isolates, and the subsequent isolates are referred to as "late" isolates (designated "E" and "L", respectively).

### Replication kinetics

**Figure 1:**
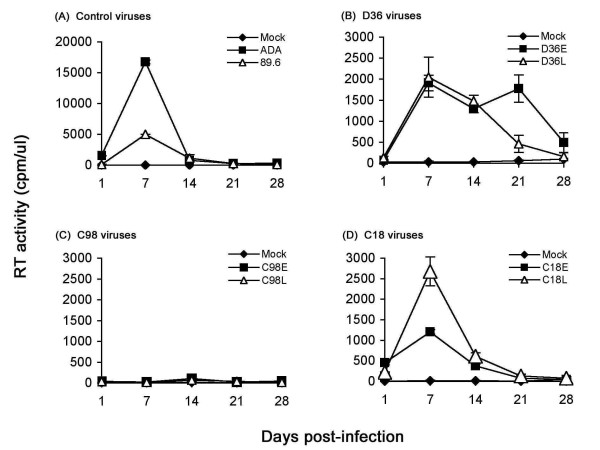
**Replication kinetics in PBMC**. PBMC were infected with equivalent amounts of each virus, as described in the Methods, and cultured for 28 days. Virus production in culture supernatants was measured by RT assays. Values shown are means from duplicate infections. Error bars represent standard deviations. Results are representative of two independent experiments using cells obtained from different donors.

We first examined the capacity of the HIV-1 isolates to replicate in PHA-activated PBMC (Fig. 1). The R5 ADA and R5X4 89.6 HIV-1 strains were included as positive controls, and replicated rapidly to high levels peaking at day 7 post-infection (Fig. 1A). Both D36E and D36L viruses replicated to comparatively low levels, with similar kinetics as ADA and 89.6 (Fig. 1B). Both C18 viruses replicated with similar kinetics, but peak levels of replication by C18L were modestly higher (approximately 2-fold) than those achieved by C18E (Fig. 1D). In contrast, replication of C98E and C98L viruses was barely detectable (Fig. 1C). Since D36 and C98 were slow progressors and C18 was a LTNP, there was no association between replication kinetics of HIV-1 isolates in PBMC and disease progression in the study subjects. However, the results highlight the heterogeneity in replication capacity by nef-deleted HIV-1 strains isolated from multiple subjects infected from a single source.

### Coreceptor usage

**Figure 2:**
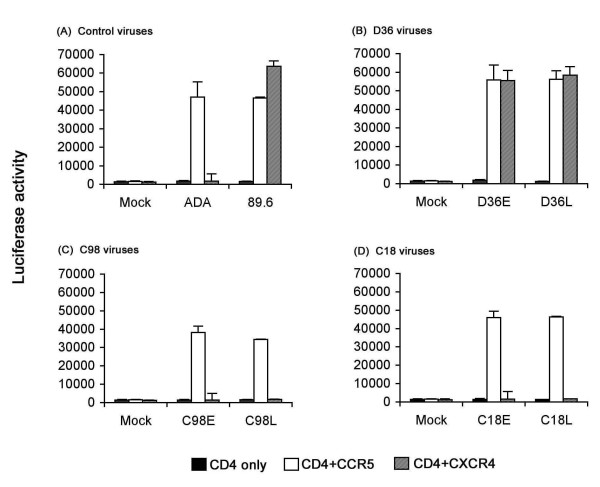
**Coreceptor usage by primary HIV-1 isolates**. Cf2-Luc cells were transfected with pcDNA3-CD4 alone or cotransfected with pcDNA3-CD4 and pcDNA3 expressing CCR5 or CXCR4 and infected with equivalent amounts of each HIV-1 isolate as described in the Methods. Cell lysates were prepared at 48 h post-infection and assayed for luciferase activity. Data are expressed as means from duplicate infections. Error bars represent standard deviations. Similar results were obtained in three independent experiments.

The results of the preceding experiments suggest the existence of additional phenotypic changes that may contribute to altered replication capacity in PBMC. We therefore compared the coreceptor usage of HIV-1 isolates in Cf2-Luc cells (Fig. 2). The R5 ADA and R5X4 89.6 strains were included as positive controls and used CCR5 or both CCR5 and CXCR4 for virus entry, respectively (Fig. 2A), as expected [9,11,36]. Consistent with results of previous studies [32], D36E and D36L viruses were dual tropic and used CCR5 and CXCR4 for virus entry (Fig. 2B). C98E, C98L, C18E and C18L viruses used CCR5 only for virus entry. Replication of dual tropic D36E and D36L viruses in PBMC was abolished by preincubation of cells with the CXCR4 inhibitor AMD3100 [37,38], but was unaffected by the CCR5 inhibitor TAK-779 [39] (data not shown). This suggests that the viral quasispecies in D36 isolates are not a mixture containing R5 variants, and confirms previous studies that showed infection of PBMC by R5X4 viruses occurs primarily via CXCR4 [40]. Thus, D36 isolates are of R5X4 phenotype, and C18 and C98 isolates are of R5 phenotype. These results indicate that the presence of a functional nef gene is not required for HIV-1 to undergo a switch in coreceptor preference from R5 to R5X4. However, since D36 harbored R5X4 variants without antiretroviral therapy while remaining asymptomatic for at least 4 years (Table 1), the results suggest that acquisition of an R5X4 phenotype is not sufficient for rapid disease progression in the absence of *nef*. Moreover, the fact that C98 maintained an R5 virus that replicated poorly in PBMC, despite evidence of disease progression, suggests that *nef*-deleted viruses may acquire increased pathogenicity *in vivo *by mechanism(s) that are not necessarily associated with changes in coreceptor usage or enhanced replicative capacity *in vitro*.

### V1V2 and V3 HTA analysis

Changes in the dominant viral quasispecies may serve to augment HIV-1 pathogenicity *in vivo *without increasing replication capacity or changing coreceptor preference *in vitro *[5]. Therefore, to determine whether distinct viral variants are present in early and late D36, C18 and C98 viruses, Env V1V2 and V3 HTA analyses were conducted.

**Figure 3:**
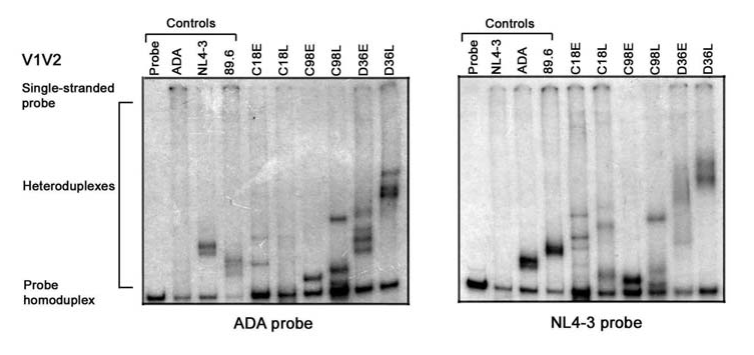
**V1V2 HTA analysis**. HIV-1 Env V1V2 regions were amplified by PCR from genomic DNA of HIV-1 infected PBMC and subjected to HTA analysis as described in the Methods. HTA analysis using a [^32^P]-labelled ADA V1V2 Env probe is shown in the left panel, and HTA analysis using a [^32^P]-labelled NL4-3 V1V2 Env probe is shown in the right panel. Similar results were obtained in three independent experiments.

**Figure 4:**
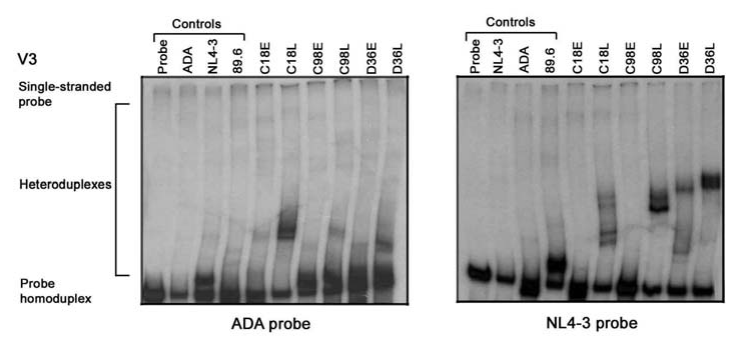
**V3 HTA analysis**. The HIV-1 Env V3 region was amplified by PCR from genomic DNA of HIV-1 infected PBMC and subjected to HTA analysis as described in the Methods. HTA analysis using a [^32^P]-labelled ADA V3 Env probe is shown in the left panel, and HTA analysis using a [^32^P]-labelled NL4-3 V3 Env probe is shown in the right panel. Similar results were obtained in three independent experiments.

The V1V2 and V3 HTAs were conducted using [^32^P]-labelled probes generated from the R5 ADA Env or the X4 NL4-3 Env (Figs. 3 and 4; left and right panels, respectively). HTA negative controls consisted of reactions containing probe alone or mixed with homologous, unlabelled target DNA to identify homoduplexes. HTA positive controls consisted of ADA probe in reactions containing unlabelled NL4-3 or 89.6 Env (Figs. 3 and 4; left panels), or NL4-3 probe in reactions containing unlabelled ADA or 89.6 Env (Fig. 3 and 4, right panels) to identify heteroduplexes. V1V2 HTAs using either probe demonstrated that C98L contained 2 dominant variants that were distinct from the single, dominant species found in C98E (Fig. 3). Similarly, V1V2 HTAs with either probe demonstrated 2 dominant variants in D36L that were distinct from 4 variants found in D36E. V1V2 HTAs using the NL4-3 probe demonstrated 3 variants in C18L that were distinct from 4 variants found in C18E. However, the ADA probe appeared to be less sensitive for detecting distinct V1V2 variants in C18E and C18L viruses. In addition to the presence of distinct V1V2 heteroduplexes between viruses isolated from individuals, V1V2 heteroduplexes were also distinct between subjects.

The V3 heteroduplex patterns also demonstrated distinct viral variants using either probe (Fig. 4). However, the resolution of V3 heteroduplexes was more readily achieved using the NL4-3 probe. V3 HTAs demonstrated 4 major variants in C18L that were distinct from a single variant present in C18E; 2 major variants in C98L that were distinct from a single major variant present in C98E; and 2 major variants in D36L that were distinct from 2 major variants present in D36E. Similar to results of the V1V2 HTAs (Fig. 3), the V3 heteroduplexes also appeared to be distinct between subjects. Together, the results of the V1V2 and V3 HTAs suggest significant inter- and intra-patient evolution of HIV-1 Env. In contrast to convergent sequence evolution previously reported for HIV-1 *nef *in the study subjects [4], the V1V2 and V3 HTA results suggest independent evolution of HIV-1 Env.

### V1V2 length polymorphism analysis

**Figure 5:**
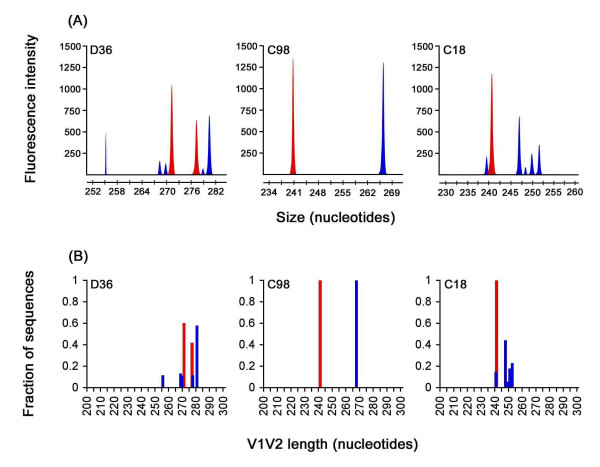
**V1V2 length polymorphism analysis**. The HIV-1 Env V1V2 region incorporating a 6-carboxy-fluorescien fluorophore was amplified by PCR from genomic DNA of HIV-1 infected PBMC and subjected to GeneScan analysis, as described in the Methods and elsewhere [41, 56]. (A) GeneScan sample files generated from amplified products. (B) Fraction of sequences with a given V1V2 nucleotide length, which was calculated from GeneScan sample files. Peaks and bars shown in red represent V1V2 amplimers from early viruses, and peaks and bars shown in blue represent V1V2 amplimers from late viruses. Similar results were obtained in two independent experiments.

The V1V2 region of HIV-1 Env contains extensive length polymorphisms, which can be utilized to compare the genetic relationships between different viral populations [41]. Furthermore, V2 region extensions have been associated with slow progression or long-term nonprogression of HIV-1 infection [23,42]. We further investigated the extent of HIV-1 Env diversity in SBBC viral isolates by measuring V1V2 length polymorphisms using GeneScan assay (Fig. 5). Although GeneScan analysis is unable to discriminate between distinct V1V2 variants of the same length which contain discrete amino acid substitutions, it has the sensitivity to detect a single nucleotide (nt) deletion or insertion within PCR products [41]. D36E virus contained 2 dominant V1V2 length polymorphisms measuring 271 and 277 nt, whereas D36L virus contained one dominant species measuring 280 nt and 4 minor species measuring 255, 268, 269 and 277 nt (Fig. 5). C98E and C98L viruses each contained single, dominant V1V2 length polymorphisms measuring 241 and 267 nt, respectively. C18E virus contained a single, dominant V1V2 length polymorphism measuring 241 nt, which was identical in nt length to the dominant species detected in C98E virus. However, C18L contained 5 distinct V1V2 length polymorphisms measuring 240, 247, 249, 250 and 252 nt. Thus, in each study subject, late viruses contained variants with V1V2 nt lengths that were distinct from those detected in early viruses.

These results suggest that significant evolution of V1V2 Env occurred in each of the study subjects, an interpretation supported also by results of the V1V2 HTA analysis (Fig. 3). That C18E and C98E viruses contained dominant variants with identical V1V2 nt length raises the possibility that these 2 subjects once harboured Env variants with some shared features. However, the increase in number of V1V2 length polymorphisms in D36L and C18L viruses compared to D36E and C18E viruses, respectively, the shift in dominant V1V2 length polymorphism in C98 viruses, and the lack of overlap between V1V2 length variants detected in D36L, C98L and C18L viruses suggests divergent evolution of HIV-1 Env in these SBBC study subjects. In contrast to previous studies [23,42], long-term survival of HIV-1 infection in these subjects was not associated with increased V1V2 nt length. Furthermore, significant increases in V1V2 nt length diversity were observed in late viruses from a SP (D36) and a LTNP (C18) compared to respective early viruses, and no increase in V1V2 nt length diversity was observed in late virus from a SP (C98); this suggests that divergent evolution of HIV-1 Env in the study subjects was neither necessary nor sufficient for disease progression.

### Sequence analysis

**Figure 6:**
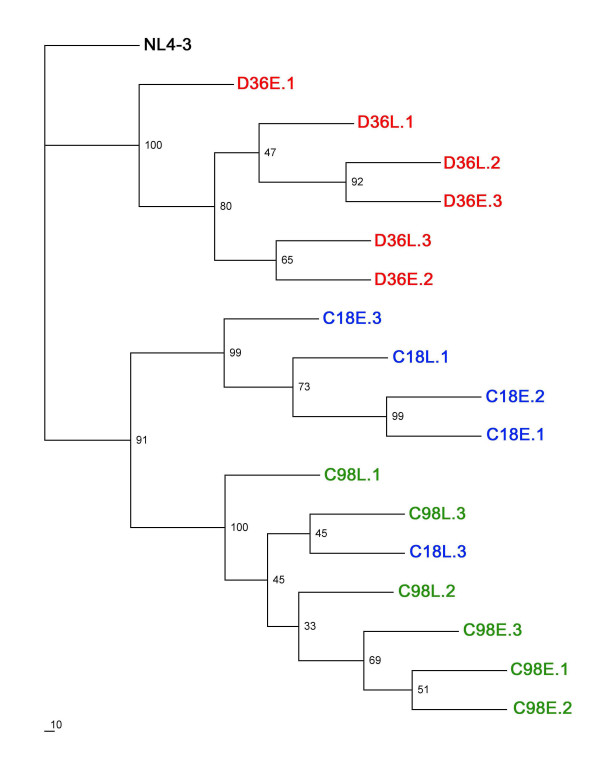
**Phylogenetic analysis**. Env nucleotide sequences were subjected to maximum likelihood analysis as described in the Methods. Branches labelled in green, blue and red represent sequences cloned from subjects C98, C18 and D36, respectively. E, clones from early viruses; L, clones from late viruses.

The preceding studies showed differences in phenotype between D36, C98 and C18 viruses and evidence of divergent Env sequence evolution. However, an association between disease progression and results of phenotypic or genetic studies could not be made. To determine the genetic basis underlying differences in viral phenotype, and to better understand Env sequence changes which may contribute to HIV-1 progression in SBBC members, the gp120 region of Env was cloned and the V1 to V3 region of multiple, independent Envs sequenced. Phylogenetic analysis of interpatient sequence sets showed monophyletic clustering of D36 Env sequences (Fig. 6). The majority of C98 and C18 Env sequences clustered separately, but 1 C18 Env (C18L.3) clustered with C98 Envs, suggesting the presence of shared sequence similarities. This is not unexpected, since C18 and C98 were infected with a closely related HIV-1 strain and, unlike D36 whose virus had a coreceptor switch (Fig. 2), both C18 and C98 continued to harbor less evolved R5 variants. Analysis of intrapatient sequence sets showed that the majority of C18E and C98E clones cosegregated separately from C18L and C98L clones, respectively. However, clear cosegregation of D36E and D36L Envs in the monophyletic D36 cluster was not evident. Since phylogenetic analysis ignores sequence insertions and deletions, this suggests that Env sequence evolution in D36 may primarily involve nucleotide insertions and/or deletions rather than discrete substitutions.

**Figure 7:**
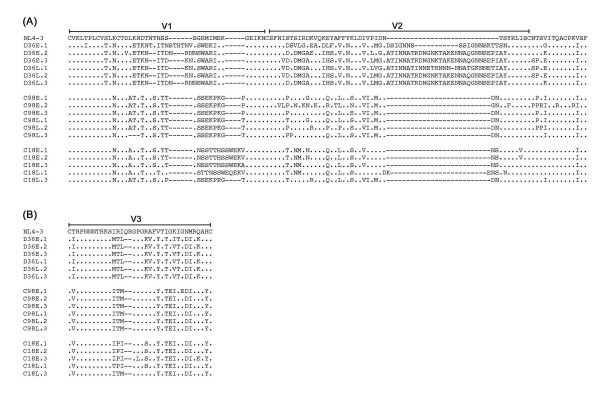
**Env V1V2 and V3 amino acid sequences**. HIV-1 Env amino acid sequences spanning the V1V2 (A) or V3 (B) regions of Env genes cloned into pGEM-T-easy were obtained as described in the Methods. Amino acid alignments are compared to Env from HIV-1 NL4-3. Dots indicate residues identical to NL4-3, and dashes indicate gaps.

Multiple sequence alignments of the V1V2 and V3 regions of Envs cloned from each virus are shown in Fig. 7A and 7B, respectively. The net charge of the V3 regions of D36E and D36L clones was +6, and the net charge of the V3 regions of C98E, C98L, C18E and C18L clones was +2 or +3. Consistent with results obtained in coreceptor usage assays with HIV-1 isolates (Fig. 2), coreceptor usage based on net V3 charge using the sinsi matrix [43] predicts D36E and D36L clones to be R5X4 phenotype, and C98E, C98L, C18E and C18L clones to be R5 phenotype. Although the presence of a basic residue at position 11 or 25 in V3 is strongly associated with CXCR4 usage [44,45], all D36E and D36L clones lacked basic residues at either position. Therefore, although D36E and D36L viruses are R5X4 phenotype in transfected Cf2th cells and use CXCR4 for HIV-1 entry into PBMC (data not shown), CXCR4 use for HIV-1 entry was not determined by the presence of charged amino acids at positions 11 or 25. Recently, the presence of isoleucine at amino acid 326 in V3, or proline or cysteine residues in V1 was shown to be important for macrophage (M) tropism of the R5X4 HIV-1 89.6 strain and other blood-derived, M-tropic R5X4 viruses [46]. In support of these results, D36E and D36L Envs lack these genetic changes, and the primary isolates replicate poorly in cultures of monocyte-derived macrophages (MDM) compared to 89.6 [47].

The base of the V1V2 stem contains a highly conserved potential N-linked glycosylation site in the CNTS sequence of NL4-3 (Fig. 7A), which is present in all but seven of 208 clade B HIV-1 Env sequences screened from the Los Alamos National Laboratory HIV Database [48]. One of 3 D36E clones and 2/3 D36L clones lacked a potential N-linked glycosylation site at this position (Fig. 7A). Similarly, the glycosylation site at this position was lacking in 1/3 C98E and 1/3 C98L clones. In contrast, the glycosylation site at the V1V2 stem was conserved among all C18E and C18L clones, and in contrast to D36 and C98 clones there was a high degree of sequence homology in this region to that of NL4-3. Elimination of a glycosylation site at this position is sufficient for CD4-independent infection by HIV-1 ADA, achieved by altering the position of the V1V2 loops and exposing the coreceptor binding site in gp120 [49-52]. Thus, alterations in glycosylation at the V1V2 stem may serve to enhance receptor binding, which could contribute to HIV-1 pathogenicity at later stages of HIV-1 infection. To this end, it is interesting to note that Env clones lacking this glycosylation site were present only in SBBC slow progressors (D36 and C98), whereas the glycosylation site was present in all Envs from the LTNP (C18). Further sequence analysis of a greater number of Env clones is required to determine the significance of this sequence change in SBBC SPs and LTNPs. In addition, further studies to biologically characterize these Envs are required to determine whether SBBC Envs exhibit functional changes that could potentially contribute to HIV-1 pathogenicity.

## Conclusion

In this study, we analyzed the phenotype and Env sequences of HIV-1 present in 3 SBBC members who were slow progressors or long-term nonprogressors. Early and late viruses from D36 were R5X4 whereas viruses isolated from C98 and C18 remained CCR5-restricted, indicating that a coreceptor switch was neither necessary nor sufficient for disease progression in these subjects. Replication capacity of these viruses in PBMC ranged from rapid to barely detectable and was not associated with disease progression. Although SBBC subjects had evidence of convergent evolution of *nef *sequence [4], analysis of Env diversity by V1V2 and V3 HTA, V1V2 length polymorphism assay, and maximum likelihood phylogeny suggest that Env sequence evolution was divergent in SP and LTNP subjects. Our results suggest that evolution toward a pathogenic Env phenotype may occur in long-term survivors infected with *nef*-deleted HIV-1, which is not necessarily associated with changes in replication capacity or coreceptor usage, or degree of Env sequence diversity.

## Methods

### Isolation of HIV-1

HIV-1 was isolated from patient's PBMC by coculture with selected PBMC according to published methods [36]. Briefly, 2 × 10^6 ^patient PBMC were mixed with 10 × 10^6 ^PHA-activated PBMC from 2 normal uninfected donors, and cocultured for 28 days in RPMI-1640 medium containing 10% (vol/vol) fetal calf serum (FCS) and 20 U/ml interleukin-2 (IL-2). Fifty percent media changes were performed twice weekly. Five million PHA-activated PBMC from a different donor were added at every second media change. Supernatants were tested for reverse transcriptase (RT) activity using [^33^P]dTTP incorporation as described previously [53]. Supernatants testing positive for RT activity were filtered through 0.45 μm filters and stored at -80°C.

### HIV-1 replication kinetics

Five hundred thousand PHA-activated PBMC were infected in 48-well tissue culture plates by incubation with 1 × 10^6 ^[^33^P] cpm RT units of virus supernatant in a volume of 250 μl for 3 h at 37°C, as described previously [32,54]. Virus was then removed, and PBMC were washed 3 times with phosphate-buffered saline (PBS) and cultured in RPMI-1640 medium containing 10% (vol/vol) FCS and 20 U/ml IL-2 for 27 days. Fifty percent medium changes were performed twice weekly, and supernatants were tested for HIV-1 replication by RT assays on days 1, 7, 14, 21 and 28 post-infection.

### Coreceptor usage

Coreceptor usage by primary HIV-1 isolates was determined using Cf2-Luc cells expressing CD4 alone, or expressing CD4 together with CCR5 or CXCR4, as described previously [32,36,54,55]. Briefly, Cf2-Luc cells were transfected with 10 μg of plasmid pcDNA3-CD4 and 20 μg of plasmid pcDNA3 containing CCR5 or CXCR4 using the calcium phosphate method, and infected 48 h later by incubation with 1 × 10^6 ^[^33^P] cpm RT units of HIV-1 in the presence of 2 μg of Polybrene (Sigma) per ml. After overnight infection, virus was removed and the cells were cultured for an additional 48 h prior to lysis in 200 μl of cell lysis buffer (Promega, Madison, Wis.). Cell lysates were cleared by centrifugation, and assayed for luciferase activity (Promega) according to the manufacturer's protocol.

### V1V2 and V3 HTA

The V1V2 probes were generated by PCR amplification of a 282 bp fragment of the HIV-1 ADA or NL4-3 Env using primers SK122 (5'-CAAGCCTAAAGCCATGTGTA-3'; corresponding to nucleotide positions 6561 to 6580 of NL4-3) and SK123 (5'-TAATGTATGGGAATTGGCTCAA-3'; corresponding to nucleotide positions 6822 to 6843 of NL4-3). The V3 probes were generated by PCR amplification of a 239 bp fragment of the HIV-1 ADA or NL4-3 Env using primers V3c (5'-CCATAATAGTACAGCTGAATG-3'; corresponding to nucleotide positions 7062 to 7081 of NL4-3) and V3d (5'-ATTTCTGGGTCCCCTCCTGAGGATTG-3'; corresponding to nucleotide positions 7276 to 7301 of NL4-3). Labelling was achieved by incorporation of α- [32P]-dCTP in the PCR, which consisted of an initial denaturation at 95°C for 5 min, followed by 25 cycles of 95°C for 30 sec, 52°C for 1 min, and 72°C for 2 min followed by a final extension step of 72°C for 7 min. Unincorporated nucleotides were removed using a QIAquick spin column (Qiagen). The V1V2 and V3 Env target DNA sequences were generated from genomic DNA of PBMC infected with each primary HIV-1 isolate by PCR using primers SK122/SK123 and V3c/V3d, respectively. Genomic DNA of PBMC infected with HIV-1 ADA, NL4-3 or 89.6 was used as controls. PCR reactions proceeded as described above, except that radiolabelled dCTP was not included. Amplimers were purified using a QIAquick spin column (Qiagen). Heteroduplex reactions were performed as described previously [21] with the following modifications. The reactions consisted of 1× annealing buffer [1 M NaCl, 100 mM Tris-HCL (pH 7.5), 20 mM EDTA], 5 μl unlabelled V1V2 or V3 target PCR product, and 2.5 μl labelled V1V2 or V3 probe. The reactions were denatured at 95°C for 4 min and then allowed to anneal on wet ice for 5 min. The heteroduplexes were separated in 5.5% (wt/vol) polyacrylamide gels in 1× Tris-borate-EDTA buffer, and were visualized by autoradiography of dried HTA gels.

### V1V2 length polymorphism analysis

V1V2 length polymorphisms in HIV-1 Env were quantified using a fluorescent-based assay that has been described in detail previously [41]. This technique measures HIV-1 sequence diversity by taking advantage of frequent length polymorphisms that occur within the V1V2 region of HIV-1 Env, and has the sensitivity to detect a single nucleotide deletion or insertion. Briefly, the V1V2 region of HIV-1 Env was amplified from genomic DNA of PBMC infected with each primary HIV-1 isolate by nested PCR using outer primers V12-51 and V12-52, and inner primers V12-50 and V12-53, as described previously [41]. The V12-50 primer used in the second round PCR was labelled with a fluorophore, 6-carboxy-fluorescien, at the 5' end (PE Biosystems). PCR amplified, fluorescently labelled products were purified using QIAquick spin columns (Qiagen), separated in 6% (wt/vol) denaturing polyacrylamide gels using an automated sequencer (ABI PRISM 377; PE Biosystems) and analysed using GeneScan software (PE Biosystems). Peaks with areas <10% of the total peak area were considered not significant, as described previously [56]. The fraction of sequences in the viral quasispecies with a given nucleotide length was calculated from GeneScan data and plotted against nucleotide length, as described previously [57].

### Env cloning, sequencing and phylogenetic analysis

A 2.1 kb fragment of HIV-1 Env (corresponding to nucleotide positions 6332 to 8452 in NL4-3) was amplified from genomic DNA of PBMC infected with each primary HIV-1 isolate by nested PCR using outer primers *env*1A and *env*1M [58], and inner primers *env*KpnI and *env*BamHI [59], as described previously [60], and cloned into pGEM-T Easy (Promega). The V1 to V3 region of 2 to 3 independent Envs cloned from each primary HIV-1 isolate was sequenced using a SequiTherm EXCEL II DNA sequencing kit (Epicenter Technologies, Madison, WI) and a model 4000L LI-COR DNA sequencer (LI-COR, Lincoln, NE). Nucleotide sequences were aligned using CLUSTALW and corrected by hand. Phylogeny was estimated by a maximum likelihood algorithm (DNAml) with a transition/transversion ratio of 2.0, empirical base frequencies, and a randomised input order of sequences. Bootstrap values were calculated from 100 resamplings of the same alignment using Seqboot.

### Nucleotide sequence accession numbers

The V1 to V3 Env nucleotide sequences reported here have been assigned GenBank accession numbers DQ665223 to DQ665240.

## Competing interests

The author(s) declare that they have no competing interests.

## Authors' contributions

LG carried out the virus replication studies, DNA sequencing and sequence analysis, and the GeneScan analyses; JS, MJC and KW carried out the HTA studies; ALC assisted with the HTA studies; MJC and JS assisted with the DNA sequencing; DAM carried out the HIV-1 virus isolations; JCL and JSS provided patient samples and clinical data; MJC, SLW, DG, ALC and DAM contributed to the study design, assisted with manuscript preparation, and helped edit the manuscript; DG undertook HIV-1 coreceptor testing in conjunction with PRG; MJC, SLW, DG, ALC, DAM and PRG analyzed and interpreted the data; PRG designed and oversaw the study, and wrote the manuscript. All authors read and approved the manuscript.
